# 24/7 TIA-service

**DOI:** 10.5334/ijic.749

**Published:** 2012-06-13

**Authors:** Frank G van Rooij, Ewoud J van Dijk, Frank-Erik de Leeuw

**Affiliations:** Department of Neurology, Radboud University Nijmegen Medical Centre, Nijmegen, The Netherlands; Department of Neurology, Radboud University Nijmegen Medical Centre, Nijmegen, The Netherlands; Department of Neurology, Radboud University Nijmegen Medical Centre, Nijmegen, The Netherlands

**Keywords:** transient ischemic attack, risk, stroke, diagnosis, acute

## Abstract

Although the symptoms of a transient ischemic attack (TIA) by definition resolve completely, the subsequent risk of cardiovascular complications is substantial. Urgent diagnosis and start of secondary prevention can reduce these risks. In light of the potential unfavourable prognosis, especially the first days after a TIA, we have developed and implemented a 24/7 TIA-service. Patients can be referred at any time and timing of analysis is determined by means of short-term risk assessment with a validated tool. Within half a day all investigations necessary to diagnose TIA and identify risk factors take place and secondary prevention at all levels is started immediately. This service is realized through integration of workflow of different specialities at multiple levels of care. Although patient service improves, the beneficial effects of a 24/7 TIA-service need to be established before further implementation is started. A preliminary analysis on the effects of continuous TIA-care and suggestions for the development of the necessary process and outcome indicators are provided.

## Introduction

A transient ischemic attack (TIA) is defined as a focal neurological deficit of sudden onset attributable to ischemia of the brain or retina which resolves completely within 24 hours. Most TIAs last less than one hour. The 24-hour cut-off point is arbitrarily, but since 1975 no new definition of TIA has been universally accepted. When symptoms last more than 24 hours, a patient is diagnosed with a cerebral infarction (ischemic stroke) [1].

Atherosclerosis is the main cause of TIAs. The major risk factors for the development of atherosclerosis and thus also for TIAs are hypertension, hypercholesterolemia, diabetes and smoking. Atherosclerosis can result in stenosis and ultimately occlusion of both large extracranial and small intracranial vessels. In large vessel atherosclerosis, such as in the internal carotid artery, thrombi can develop on the (post-)stenotic vessel surface, detach and be transported upstream until they occlude an artery and lead to cerebral ischemia. Thrombi can also develop in the heart as a result of atrial fibrillation or a prior myocardial infarction, which are other major causes of TIAs.

Although the symptoms of a TIA, by definition, completely resolve, it is by no means a benign condition. The short and long-term risks of cardiovascular complications, such as cerebral and myocardial infarction are markedly increased after a TIA. In high-risk patients, the risk of subsequent cerebral infarction in the first two days after a TIA is 10% [2]. Of all TIA patients, 10% develop a cerebral infarction within 90 days [3, 4]. The annual risk of myocardial infarction is doubled after a TIA [5]. The combined risk of cerebral infarction, myocardial infarction and death within 12 months after a TIA is 22% [4].

Of all patients with a cerebral infarction, 10% have had a TIA prior to the infarction [6]. This illustrates that a TIA can be considered a cerebral infarction warning signal. As such, it provides a window of opportunity for intervention that can, if implemented with considerable speed, reduce the risk of brain infarction and subsequent major disability. For this reason, a TIA should be considered a medical emergency. Urgent diagnosis of risk factors involved and intervention when needed has been proven beneficial [7, 8]. Unfortunately, daily common practice shows that this procedure often takes place several days after referral with potentially deleterious effects. In light of the potential unfavourable prognosis, especially the first days after a TIA, we have developed and implemented a 24/7 TIA-service at the Radboud University Nijmegen Medical Centre (RUNMC).

## TIA-service pathway

### Basic principle

The basic principle of the 24/7 TIA-service is to assess the short-term risk of stroke and determine the timing of analysis accordingly. The short-term risk is estimated with a validated tool, the ABCD^2^ risk score (Table 1) [2]. By means of evaluating five factors (age, blood pressure, clinical symptoms, duration of symptoms and diabetes) a sum score between 0 and 7 is obtained, with higher scores corresponding with higher short-term risks of cerebral infarction. Patients with scores of 6 or 7 are generally considered high-risk patients who need immediate evaluation. Further refinement of this prognostic rule by adding other clinical and imaging factors is on its way, but needs further validation.

### Patient referral

When a general practitioner suspects a patient of having had a TIA, a neurologist from the RUNMC can always be contacted. Moreover, a special telephone number has been installed, enabling contact with a specialized vascular neurologist 24/7. The neurologist consulted determines the speed and location of further analysis through the use of two simple questions:

- Have the symptoms completely resolved?
- If so, what is the risk of a subsequent stroke?

When there is doubt about the resolution of symptoms or when the risk of subsequent stroke is high, determined by an ABCD^2^ risk score of >5, the patient is referred to the emergency department for immediate analysis. Furthermore, patients with recurrent TIAs in a short period of time and those known with atrial fibrillation are referred as well.

### Diagnosis

The diagnosis of TIA can be difficult given the fact that symptoms often are no longer present by the time a patient is seen. Taking a careful history is therefore the cornerstone of the diagnosis, paying special attention to items, such as an acute onset of symptoms, combinations of symptoms suggestive of a specific cerebral vascular territory and cardiovascular risk factors. A neurological examination is performed to ensure that symptoms have completely subsided. Additional investigations can increase the likelihood of TIA. Cerebral magnetic resonance imaging (MRI) including diffusion weighted imaging can detect recent ischemia in a minority of TIA-patients [9]. Other additional investigations are performed in order to identify possible causes of TIA with special attention to cardiac arrhythmias, hypertension, hypercholesterolemia, stenosis of the cervical arteries and lifestyle factors, such as smoking habits and overweight.

### Treatment

The aim of TIA treatment is prevention of new TIAs, cerebral infarction and myocardial infarction. In general it consists of antithrombotics, a lipid lowering and an antihypertensive drug. In case of a suspected cardiac origin of the TIA (most often atrial fibrillation), anticoagulant therapy is started and the cardiologist is involved in further patient treatment. When significant stenosis of the cervical arteries is the probable cause of TIA, a vascular surgeon is consulted and, when deemed necessary, fast-track carotid endarterectomy is performed. All these treatments have been proven to reduce the risk of subsequent vascular events [10, 11].

### TIA-service

Within half a day all investigations necessary to diagnose TIA and identify possible risk factors take place. Results are immediately discussed with the patient, secondary prevention is started and appropriate referrals to other specialists are arranged. Finally, short-term follow-up at the outpatient department is planned. The programme consists of the following:

- Welcome and information about the program by acute day ward nurse;
- History taking and physical examination by neurologist;
- Length and weight measurements, determination of body mass index;
- Laboratory investigations consisting of kidney and liver functions, full blood count and fasting glucose and cholesterol profile;
- Urine examination for proteinuria;
- Electrocardiography with special attention to atrial fibrillation, cardiac ischemia and left ventricular hypertrophy. In case of doubt, immediate consultation of a cardiologist is possible;
- Standardized repeated blood pressure measurements;
- Diagnostic imaging, either MRI or computed tomography (CT) of brain, cervical and intracranial arteries;
- In young patients (<50 years) additional investigations are done, since aetiology often differs from older patients.
- Neurologist discusses the results with the patient. Information is provided about the disease, its risk factors, secondary preventive medication and life style risk factors and advice what to do in case of recurrence. Finally a personalized follow-up is scheduled during which ample attention is given to compliance of medication, but also to lifestyle risk factors (stop smoking, healthy food and exercise). Thereafter the patient returns home.

Diagnostic imaging of the brain is preferentially performed with MRI, since this enables the possibility of detecting recent ischemia. Daily fixed slots at the radiology department are available. When there are contraindications for MRI or no slots are available any more, CT of the brain and cervical arteries is performed, with the aid of an iodine-containing contrast agent. When there are contraindications for the latter, duplex ultrasound of the cervical arteries is added to CT of the brain.

A neurologist as well as a radiologist evaluates all diagnostic imaging. The radiologist makes an official report within minutes and actively reports unexpected or acute abnormalities to the responsible neurologist. Attention is especially focused on signs of recent or old ischemia, other structural abnormalities (for example tumours, haemorrhages) as well as anatomy and stenosis of the cervical and cerebral vasculature.

When the TIA-service takes places at the emergency department instead of the acute day ward, the protocol and sequence of investigations is essentially the same. However, for logistic reasons, there are a few minor differences:

- Determining fasting glucose and cholesterol values is frequently not possible, since patients have often already eaten when seen at the emergency department. These are postponed to the follow-up visit at the outpatient department;
- Diagnostic imaging of brain and cervical arteries always consists of CT including angiography with an iodine-containing contrast agent. The latter is substituted by duplex ultrasound when contraindications are present and in case of significant cervical artery stenosis additional MR angiography is planned for the next day.

Through combining history and additional investigations, the final judgement of whether a TIA has occurred is made. When no TIA has taken place, further follow-up at the general neurology outpatient department is planned when deemed necessary. When the diagnosis of TIA is made, secondary prevention is started immediately. This rests on three cornerstones. The first is secondary prevention through medication, consisting of a combination of antithrombotics or anticoagulant therapy, depending on the suspected cause of the TIA. Furthermore a lipid-lowering drug is added when the value of fasting low-density-lipoprotein cholesterol is >2.5 mmol/l (96.7 mg/dl) and antihypertensive medication is started when blood pressure is >130/80 mmHg. The second cornerstone is carotid surgery when necessary and is proven beneficial to patients with an ipsilateral carotid artery stenosis >70%. In these cases the vascular surgeon is consulted immediately in order to accomplish surgery as soon as possible, but at least within 14 days after the TIA. The third cornerstone is evaluation of the lifestyle and assessing the motivation of a patient to for example stop smoking, increase exercise and change eating habits. This is organized in a structured way, through a multidisciplinary outpatient clinic organized by a specialized nurse. Based upon a hospital-wide protocol (developed by cardiologists, vascular internists, neurologists and vascular surgeons) lifestyle and cardiovascular risk factors are identified. Patient motivation is determined through a structured questionnaire and motivated patients are offered lifestyle interventions, provided by specialized nurses.

### Patient information

The visit to the TIA-service ends with an extensive conversation between patient and neurologist, during which the concept of TIA is explained. Possible side effects of the medication and consequences for driving abilities are discussed. A self designed brochure containing all this information is given along with each patient. Finally, instructions are provided on how to act in case of recurrent symptoms.

### Follow-up

Patients are contacted by telephone two weeks after the initial visit by their vascular neurologist and are seen at the multidisciplinary outpatient clinic four weeks later. Afterwards, consultation is continued at regular intervals for different lengths of time, depending on the specific situation of a patient. The general practitioner is kept informed through letters and is actively involved in the treatment.

## Analysis

A nationwide evaluation of TIA care in the Netherlands has not been performed until now. We therefore assessed the effect of the introduction of the 24/7 TIA-service by comparing different variables since the start of this service.

The 24/7 TIA-service started during 2010 and data on patient’s and doctor’s delay became available right after the introduction of the 24/7 TIA facility. During the first full year of operation the total time from symptoms to diagnosis decreased by 30% from just over 10 days to 7 days. Unfortunately, most of these days were due to patient’s delay, whereas doctor’s delay was usually less than 48 hours on average.

In order to accurately determine the effect of a 24/7 TIA-service on delay to analysis and start of secondary prevention, a longer evaluation period is needed. Nevertheless, the currently available data do seem to point towards a reduction in both doctor’s and patient’s delay. This can potentially prevent stroke and other cardiovascular events that are associated with a high risk in the first days and weeks after a TIA. This is of course the ultimate goal of an around-the-clock TIA-service. However, a nationwide analysis of patient data will be needed to perform an adequately powered study of the risk reducing effects of this care pathway.

## Discussion

### Evaluation

Although international studies have shown a beneficial effect of early diagnosis and treatment of TIAs with a reduced number of subsequent cerebral infarctions [7, 8], there is no such evidence for the Dutch situation. It is as yet unclear whether a costly and labour intensive service, such as around-the-clock TIA-care would improve the final outcome for TIA-patients. Furthermore, if it does improve outcome, it is uncertain whether this holds true for all TIA patients.

In the Netherlands, general practitioners (GPs) function as a gatekeeper to further specialized care. Patients experiencing a medical problem, consult their GP for advice and, if deemed necessary, are sent to a specialist for further analysis and treatment. Alternatively, patients suffering form an acute problem can turn to an emergency ward (ER), where they are primarily seen by an ER-doctor, who can decide to consult a specialist.

This organisation of care has its benefits and limitations. With respect to a TIA, and the fact that this should be treated as a stroke, this situation is prone to doctor’s delay. Offering a 24/7-TIA service does not directly change the GP or ER-physician neurological evaluation interval. However, when a patient is finally referred to a neurologist for analysis of a possible TIA, any delay in this referral is completely ruled out.

The main reason of delay between TIA symptoms and specialised analysis is patient’s delay. The most important way to reduce this delay is education about TIAs and their consequences. This is independent of the presence of a 24/7 TIA-service. However, some patients might be reluctant to immediately seek medical care after a TIA, because they know it will take days before they are eventually analysed and any intervention takes place. Knowing, for instance through media attention, that an around-the-clock dedicated TIA-service exists, where a patient is immediately fully examined and interventions can be started right away, could possibly help patients to decide to seek immediate medical attention. This is the category of patients that might benefit the most from an around-the-clock TIA-service.

A theoretical beneficial effect on the number of strokes and myocardial infarctions has to be proven before our patient-friendly, but also more costly service is implemented in other centres. When proven, we should set out to determine the crucial steps in the process. In order to determine these steps the process of care around the diagnosis and treatment of TIAs in the Netherlands has to be evaluated. The primary end point would be the number of cerebral infarctions and myocardial infarcts occurring shortly after a TIA. In order to monitor the process a combination of outcome and process indicators has to be developed, making it possible to relate possible variations in outcome to changes in the process of care.

### Indicators

The main outcome variable and quality indicator of the process of care around TIA is the incidence of cerebral infarction after a TIA. Both the EXPRESS-study [7] and the SOS-TIA Trial [8] have demonstrated that swift analysis and treatment of TIA can reduce the number of subsequent cerebral infarctions by 80%. Furthermore the EXPRESS-study showed a clear reduction in costs through a decrease in the number of hospital admissions, acute costs and disability after six months [12]. Therefore, both short-term and long-term costs can function as an outcome measure in the evaluation of the process of care around TIA.

In the process of care around TIA there are many aspects that can serve as a process indicator. Although evaluation at the emergency department can take place at any time of day, the inherent commotion there can affect the duration of the visit. Waiting times before a neurologist sees a patient, before diagnostic imaging is performed or before results are discussed can increase substantially. These waiting times can function as process indicators. For the TIA-service at the acute day ward the same indicators can be used. The aim of the 24/7 TIA-service is to diagnose and treat as soon as possible. A valid indicator of the logistics of the process would be the proportion of patients analyzed within 24 hours. Ideally, this is more than 90%. For this purpose the exact times of the TIA, the subsequent visit to the general practitioner and referral are recorded at the 24/7 TIA-service.

A neurology resident supervised by a vascular neurologist executes the 24/7 TIA-service. Care around a TIA patient is based on a clear protocol, increasing the level of expertise. This is the case not only for the neurology, but also for the radiology department, where specialized supervision is available. Due to the standardized structured approach patient volume is ever increasing with an attendant increase of expertise by the various health professionals. The personalized secondary prevention and possible life style modification is carried out under the responsibility of a specialized nurse. The secondary prevention is personalized in such a way that all risk factors and cardiovascular disease are discussed on a patient-by-patient basis during a multidisciplinary consultation between neurologist, cardiologist, vascular internist and vascular surgeon. This personal advice, both in terms of medication and life style can be executed in conjunction with both the first (general practitioner) and second line of care (specialized care). In order to implement a 24/7 TIA-service, recent changes in work structure had to take place. The level of expertise can therefore serve as an index-specific indicator in the analysis of changes in the process of care.

### Realization

Given the complexity of the organization in an academic medical centre, realizing the 24/7 TIA-service took place rather smoothly. There is clear evidence that a round-the-clock mode of operation works, which provides a base of evidence on which to perform changes in the work structure. Furthermore, during weekly scientific meetings the relevant publications have been discussed with the neurologists and residents, questioning themselves whether this system should be implemented in our hospital. Because of the evidence-based medicine attitude, every neurologist was convinced that a 24/7 facility would increase the level of patient care. Moreover, the concept of a 24/7 TIA-service emphasises the urgency for medical attention, possibly increasing the sense of emergency in the general population.

### Future

Implementing 24/7 TIA-services in the Netherlands should only be stimulated when there is clear evidence that such a procedure indeed leads to a better outcome, which is a reduction in the number of cerebral infarctions. The increased costs associated with this service can only be accounted for when it is shown to lead to a reduced disease burden. Not only the immediate higher costs of the service should be taken into account, but also the possible decrease in long-term costs, for instance due to less or shorter hospital admissions, less frequent acute interventions with emergency visits and transport, and reduction of the number of severely disabled patients with all associated costs. Such a cost-benefit analysis is still missing, since 24/7 TIA-services have not been implemented elsewhere in the Netherlands.

Implementing 24/7 TIA-care throughout the Netherlands relies on cooperation of health care professionals at different levels. Neurologists, radiologists, cardiologists, internists and vascular surgeons will have to accommodate their working routine to fast and flexible analysis and treatment. Specific training on vascular neurology can be necessary. Nursing staff, radiologic laboratory staff and outpatient department personnel are all part of the chain of care around TIA patients. To optimize care at local as well as regional and national level money, personnel and infrastructure are needed.

Patients should be aware of the signs and symptoms of a TIA in order to search medical attention in time. General practitioners will also have to be informed of the necessity and possibility of swift analysis and intervention, so that acute referrals actually take place.

Implementing 24/7 TIA-care will put a strain on health care professionals at every level. Therefore it is of the utmost importance to unequivocally establish the benefit of such a service before continuing to a national implementation. In this there lies an important role for neurologists, patient associations and health insurance companies.

## Conclusion

24/7 TIA-service provides an improvement in both patient service and outcome. Implementation took place through the integration of workflow of different specialities and the design of new work modes aimed at flexibility and speeds. The motivation for these changes was the evidence available in literature that around-the-clock care for TIA patients works. It both improves the short-term outcome and possibly reduces the associated long-term costs.

A preliminary analysis of the effect of this service on delay from TIA to start of secondary prevention shows a reduction in this delay. However, this is but a surrogate marker for the ultimate goal, namely a reduction in the incidence of cardiovascular events after a TIA. Such an effect of an around-the-clock TIA-service has to be established before implementation throughout the country takes place. Indicators, both process and index-specific are needed to relate a possible change in outcome to the changes in workflow. The combination of changes in patient care and workflow, implementation of a new mode of work and the design and monitoring of indicators necessitates the integration of care throughout every level in the chain of care, from patients and general practitioners to medical specialists and managers to ultimately health insurance companies and political decision makers.

## Figures and Tables

**Table 1.**
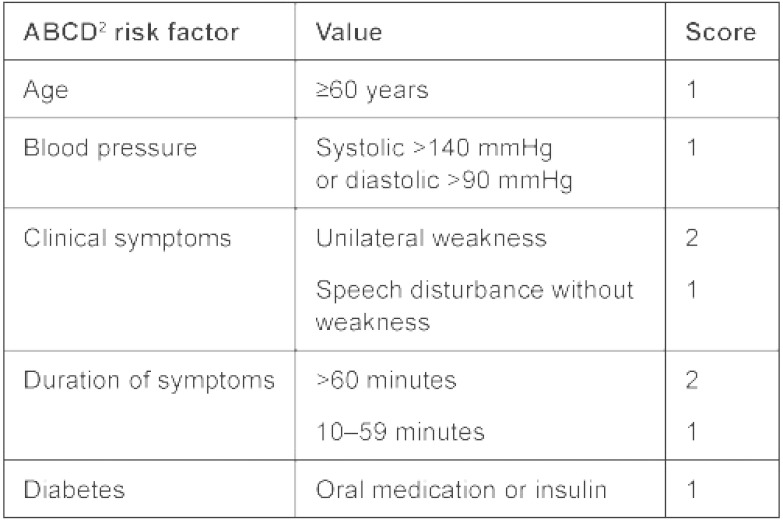
ABCD^2^ risk score

## Reviewers

**Martien Limburg**, Prof. Dr., MBA, neurologist, Flevoziekenhuis, Almere, The Netherlands

**Renee F. Lyons**, Ph.D, Bridgepoint Chair in Complex Chronic Disease Research TD, Scientific Director Bridgepoint Collaboratory for Research and Innovation, Professor Dalla Lana School of Public Health and Institute of Health Policy, Management and Evaluation, University of Toronto Bridgepoint Health, Ontario, Canada

**Giuseppe Micieli**, Direttore Dipartimento di Neurologia d'Urgenza IRCCS Istituto Neurologivo Nazionale C Mondino, Pavia, Italy
